# Strategietreffen: Virushepatitis in Deutschland eliminieren – Was ist zu tun?

**DOI:** 10.1055/a-1978-9021

**Published:** 2023-02-03

**Authors:** T. L. Tergast, U. Protzer, S. Zeuzem, L. Heitmann, C. Sarrazin, M. Lehmann, P. Ingiliz, M. Cornberg, R. Zimmermann, M. G. Gerlich, P. Buggisch, B. Wiebner, H. Wedemeyer

**Affiliations:** 1 Klinik für Gastroenterologie, Hepatologie und Endokrinologie, Medizinische Hochschule Hannover, Carl-Neuberg-Str. 1, 30625 Hannover; 2 Institut für Virologie, Technische Universität München/Helmholtz Zentrum München, München.; 3 Medizinische Klinik I Gastroenterologie, Hepatologie, Pneumologie, Endokrinologie, Universitätsklinikum Frankfurt, Frankfurt, Deutschland.; 4 Mitglied des deutschen Bundestages, Platz der Republik 1, 11011 Berlin; 5 Medizinische Klinik II, Innere Medizin, St. Josefs-Hospital Wiesbaden, Germany; 6 Justizvollzugskrankenhaus JVA Plötzensee, Saatwinkler Damm 1A, 13627 Berlin, Germany; 7 Hôpitaux Universitaires Henri Mondor; 8 Deutsche Leberstiftung, Hannover, Germany; 9 Abteilung für Infektionsepidemiologie, Robert Koch-Institut, Berlin, Germany; 10 Bundeszentrale für gesundheitliche Aufklärung, Köln; 11 ifi-Institut für interdisziplinäre Medizin, Hamburg; 12 Hepatitis B and C Public Policy Association (HepBCPPA)

## Virushepatitis – ein (un-)lösbares Problem?

Chronische Virushepatitiden stellen nach wie vor ein globales gesundheitliches Problem dar. Weltweit zählen insbesondere die chronische Hepatitis B (HBV)-Virusinfektion und Hepatitis C (HCV)-Virusinfektion zu den Infektionserkrankungen, die kumulativ für die meisten Todesfälle verantwortlich sind und einige Schätzungen vermuten einen weiteren Anstieg der Todesfälle bis 2040 1 . Beide Erkrankungen sind heutzutage gut behandelbar, für die HBV-Infektion existiert ein gut wirksamer Impfstoff und eine HCV-Infektion ist in der Regel mit neuen direkt-antiviralen Medikamenten dauerhaft heilbar 2 3 . Die Weltgesundheitsorganisation (WHO) hat im Jahre 2016 Eliminationsziele festgelegt, um eine Bedrohung der öffentlichen Gesundheit durch Virushepatitiden zu adressieren. Für Europa hat die Hepatitis B & C Public Policy Association, ein Verein mit dem Ziel der Bekämpfung viraler Hepatitiden in der EU, bereits mehrfach Strategietreffen abgehalten, um Herausforderungen auf dem Weg zur Elimination zu erkennen, zu analysieren und zu bekämpfen sowie den internationalen Austausch von Ideen und Programmen zu fördern 4 5 . Am 09.02.2022 hat die Deutsche Leberstiftung – in Kooperation mit der Hepatitis B & C Public Policy Association ebenfalls ein Treffen durchgeführt, um auf nationaler Ebene den Austausch der verschiedenen Akteure wie Patientenvereinigungen, Ärzt*innen, Wissenschaftler*innen und Politiker*innen zu fördern.

## Wo stehen wir?

Im Gegensatz zu anderen europäischen Ländern sind die HBV- und HCV-Prävalenzen in Deutschlands Bevölkerung vergleichsweise gering. Im Hinblick auf HBV beträgt die Prävalenz aktiver Infektionen in der Gesamtbevölkerung zurzeit 0,3–0,7 %, bei der HCV-Infektion sind es 0,2–0,4 % 6 7 . Je nach betrachteter Bevölkerungsgruppe gibt es jedoch deutliche Unterschiede. Bei Migrant*innen wird eine erhöhte HBsAg-Prävalenz von 2,3–3,6 % berichtet 6 . Inhaftierte weisen in zurückliegenden Studien eine HCV-Prävalenz von 14,3 % auf 8 9 . Aktuelle Daten für Inhaftierte sind nicht verfügbar. Weiterhin geht aus vorläufigen Analysen einer Prävalenzstudie in Berlin hervor, dass bei ca. 16 % der teilnehmenden Wohnungslosen eine aktive HCV-Infektion vorliegt 10 . Eine weitere Risikogruppe stellen Männer, die Sex mit Männern haben (MSM), dar. So wurde zuletzt in einer Kohorte von HIV-infizierten MSM eine, im Vergleich zur Normalbevölkerung deutlich erhöhte, HCV-Prävalenz von 3,3 % angegeben 11 . Das höchste Risiko für eine Infektion haben weiterhin Menschen mit injizierendem Drogenkonsum (engl. Abk. PWID): Die DRUCK-Studie berichtet Prävalenzen zwischen 23–54 % von aktiver HCV-Infektion und 5–33 % im Hinblick auf HBV 12 . Chronische HBV-Infektionen lagen bei 0,3–2,5 % der Untersuchten vor 13 . Für den Nachweis von HBV- und HCV-Infektionen gilt eine Meldepflicht für Labore, für akute Virushepatitiden auch für Ärzt*innen 14 . Auch in Anbetracht der gemeldeten Infektionsrisiken bei HCV-Neudiagnosen im Jahr 2020 ist insbesondere der intravenöse Drogenkonsum mit über 60 % der größte identifizierbare Risikofaktor für eine neu übertragene HCV 15 .

Aktuelle Zahlen über die Gesamtzahl von infizierten Menschen in Deutschland liegen zurzeit faktisch nicht vor. Vereinzelt gibt es Modellierungsstudien, jedoch fehlt es an „Real-World“-Daten 16 . Die WHO hatte in ihren initialen Eliminationszielen eine relative Änderung aktueller Infektionszahlen vorgeschlagen. Jedoch ist dies für Länder mit niedriger Prävalenz nur erschwert zu erreichen, weshalb die Eliminationsziele in diesem Jahr auf absolute Zahlen geändert werden könnten 17 . Im Jahre 2022 sollen so die Eliminationsziele angepasst werden. Bei allgemein niedrigen Inzidenzen in der Gesamtpopulation hat Deutschland seine Ziele teilweise bereits erreicht. Allerdings besteht mit sehr hoher Wahrscheinlichkeit ein gravierendes Defizit, was Hochrisikogruppen angeht: Ein Interimsziel bis 2025 ist die 80 %ige Senkung der HCV-Prävalenz bei PWIDs. Insgesamt besteht hier jedoch ein Mangel an Daten sowie an zielgruppenspezifischen Programmen zur Bekämpfung der Virushepatitis bei PWIDs, sodass nicht davon auszugehen ist, dass das Ziel bislang auch nur ansatzweise erreicht wurde.

## Probleme und Lösungen

Im Oktober 2021 wurde durch ein Screening auf HBV- und HCV-Infektionen eine wichtige Ergänzung im Rahmen der „Gesundheitsuntersuchung“ (vormals „Check-up 35“) vorgenommen, um bisher nicht detektierte Infektionen festzustellen. Aus ersten Daten von über 4.700 Teilnehmenden geht hervor, dass bei 0,4 % eine neue HBV-Infektion und bei 0,1 % eine neue HCV-Infektion entdeckt wurde 18 . Zirka 40–50 % der Bevölkerung nehmen die Gesundheitsuntersuchung wahr, jedoch zeigen Studien, dass insbesondere Menschen mit niedrigerem sozioökonomischem Status tendenziell weniger an einem allgemeinen „Check-up“ teilnehmen 19 . So ist zu erwarten, dass insbesondere Risikogruppen im neu eingeführten Screening unterrepräsentiert sind. Das bedeutet einerseits, dass Maßnahmen notwendig sind, um mehr Menschen auf die Gesundheitsuntersuchung aufmerksam zu machen. Andererseits müssen auch die Hausärzt*innen motiviert werden, an die Gesundheitsuntersuchung zu denken und diese zu initiieren. Die Hausärzt*innen stellen eine wichtige Ressource auf dem Weg zur Hepatitis-Elimination dar und dürfen nach erfolgter Diagnose antivirale Behandlungen durchführen. Allerdings gibt es immer wieder Ängste vor Regressen durch die Krankenkassen bei insgesamt aktuell noch hohen Medikamentenkosten 20 . An dieser Stelle sei klar festgestellt, dass eine indikationsgerechte antivirale Therapie keine Grundlage für einen Regress bietet.

Wie oben bereits erwähnt, existiert im Gegensatz zur HCV-Infektion eine wirksame Impfung zur Prävention einer HBV-Infektion. Diese wird seit 1995 durch die ständige Impfkommission (STIKO) für Säuglinge und Kleinkinder empfohlen, nicht zuletzt, da eine Infektion in diesem Alter mit einer hohen Chronifizierungsrate assoziiert ist. Leider belief sich die Inanspruchnahme der Impfung im Jahre 2019 zum Zeitpunkt der Schuleingangsuntersuchung mit großen Schwankungen zwischen den Bundesländern zwischen 78,9 % und 94,5 % 21 . Eine Förderung von Impfkampagnen sowie Aufklärungsarbeit können hier die Impfbereitschaft erhöhen.

Insbesondere Risikogruppen wie Migrant*innen, Inhaftierte, MSM oder PWIDs sind bereits einem hohen Maß sozialer Ungleichheit und Stigmatisierung ausgesetzt, welche durch eine chronische Infektion mit einem Hepatitis-Virus weiter aggraviert werden kann 22 . Ein zentrales Problem ist neben mangelnder Aufklärung der Risikogruppen ein oftmals fehlender direkter Zugang zum Gesundheitssektor. Der Abbau von Barrieren ist daher wichtig, beispielsweise, indem niedrigschwellige Test- und Behandlungsangebote geschaffen werden. Weiterhin sind insbesondere Drogengebrauchende keine einheitliche Gruppe, da es viele unter ihnen gibt, die trotz ihrer Suchterkrankung einen strukturierten Tagesablauf, eine tägliche Arbeit und feste soziale Strukturen haben 23 . Nach aktuellen Schätzungen ist in Deutschland mehr als die Hälfte der Menschen mit Opioid-Abhängigkeit in einer Substitutionsbehandlung 24 . Der regelmäßige Kontakt mit dem medizinischen System könnte besser genutzt werden, um Substituierte regelmäßig zu testen, zu impfen sowie diagnostizierte Infektionen zu behandeln. Viele der aktiv drogengebrauchenden Menschen besuchen regelmäßig niedrigschwellige Einrichtungen der Drogenhilfe wie Kontaktläden oder Drogenkonsumräume. Hier könnte mit niedrigschwelligen Testangeboten ein kosteneffizienter Ansatz geboten werden, um auch Menschen, die einen unzureichenden Zugang zur Regelversorgung haben, zu erreichen. Allerdings gibt es auch Menschen mit chronischer Hepatitis, die derartige Angebote nicht wahrnehmen und als „schwer zu erreichen“ gelten. Insbesondere für diese Gruppe gibt es sogenannte Mikro-Eliminations-Strategien, also gezielte örtliche und zeitliche Interventionen in einer bestimmten Bevölkerungsgruppe, um mit wenig Ressourcen viele Menschen zu erreichen, zu testen und zu behandeln 4 25 26 . Ein Beispiel einer Mikro-Eliminations-Strategie kann eine mobile Diagnostik- und Therapieeinheit mit speziell geschultem Personal sein, die medizinische Dienste zu den Menschen einer Risikogruppe bringt – von Beratung bis Therapie. Dies ist ein konkreter Weg, um Barrieren abzubauen und die medizinische Versorgung von Risikogruppen zu verbessern und wurde bereits in einigen europäischen Ländern erfolgreich eingesetzt 26 . An dieser Stelle sei noch betont, dass der Link zu einer Behandlung nach erfolgter Diagnose einer Hepatitis nicht immer gelingt 27 28 . Diese Lücken in der Versorgungsstruktur wurden bereits häufig aufgezeigt und bestehen nicht nur bei Risikogruppen, sondern auch in der Normalbevölkerung. Es existieren bereits Modelle und Vorschläge, welche eine Art „One-Stop-Shop“ mittels Point-of-care-Diagnostik und anschließender antiviraler Therapie möglich machen 29 . Jedoch sind diese Modelle weit davon entfernt, flächendeckend eingesetzt zu werden. Hier bedarf es entsprechender finanzieller Förderung sowie politischen Willens.

Weiterhin stellen Inhaftierte eine Risikogruppe dar, v. a. da Drogenkonsum durch den Verstoß gegen das Betäubungsmittelgesetz und Beschaffungskriminalität zu häufiger Inhaftierung führt und sich somit die Gruppen überschneiden. Die Haft kann jedoch auch als Chance angesehen werden, um mit Beratungs- und Therapieangeboten vor Ort eine Vielzahl an Betroffenen zu erreichen. Aktuell fallen Inhaftierte noch vielerorts aus dem Versorgungsangebot heraus, da die Krankenversicherungen nicht zuständig sind. Eine medizinische Versorgung in Haft ist gegeben. Allerdings sind HCV-Therapien nicht flächendeckend verfügbar. Es gibt einige Haftanstalten, die längst alle ihre Patient*innen behandeln und andere Zentren, die wegen unzureichender Ressourcen keine Behandlungsangebote anbieten können. An dieser Stelle wäre es wichtig, dass in der Vollzugsmedizin eine bundeseinheitliche Vorgehensweise erarbeitet wird, um im gesamtgesellschaftlichen Interesse eine HCV-Elimination zu erreichen. Es braucht den politischen Willen, um Maßnahmen zur Gewinnung und Förderung von personellen Ressourcen im Vollzug zu stärken, gerade auch um einen diskriminierungsfreien Vollzug zu gewährleisten 30 31 .

Zudem ist festzuhalten, dass, selbst nach Ausheilung, das Risiko für eine Reinfektion bei einer HCV-Infektion besteht, insbesondere bei MSM 32 . Im Hinblick auf MSM zeigte sich zuletzt eine Abnahme der HCV-Prävalenz. Allerdings wurde bereits mehrfach festgestellt, dass diese Gruppe nach erfolgreicher HCV-Behandlung ein hohes Risiko für eine HCV-Reinfektion aufweist 11 33 . Das Risiko für eine Reinfektion in dieser Gruppe überstieg selbst das in der Gruppe der PWIDs. Insbesondere Menschen mit hoher Viruslast zeigen detektierbare Konzentrationen von HCV-RNA nasal sowie rektal auf, was ein Risiko für eine erneute HCV-Transmission bei Hochrisiko-Sexpraktiken sowie dem Teilen von „Schnupfbesteck“ birgt 34 35 . Es resultiert demnach aus dem hohen Reinfektionsrisiko ein besonderer Bedarf an präventiven Maßnahmen sowie Aufklärungsmaßnahmen. Insgesamt ist nicht nur bei MSM, sondern bei allen Menschen mit durchgemachter HCV-Infektion auf eine adäquate Aufklärung und Nachsorge der Menschen zu achten, um einen langfristen und nachhaltigen Therapieerfolg zu erzielen. Zudem müssen flächendeckende Strukturen auf- und ausgebaut werden, die dazu beitragen Infektionsrisiken abzubauen, wie z. B. Ausweitung von Opioid-Substitutionsbehandlungen und Zugang zu sterilen Konsumutensilien für Menschen, die Drogen gebrauchen, auch in Haftanstalten.

### Fazit

Die Mittel für eine effektive Bekämpfung von Hepatitiden sind etabliert. Es fehlt jedoch an Ressourcen und Daten, um eine nachhaltige Elimination von HBV und HCV zu gewährleisten. Im Rahmen des Strategietreffens wurden somit folgende Punkte festgehalten:

- Die Erkennung und Elimination der Hepatitis-Virusinfektionen muss eine öffentliche Gesundheitspriorität sein und sollte adäquat finanziert werden.
- Daten hinsichtlich der genauen Infektionszahlen sind nur begrenzt vorhanden, eine Aktualisierung epidemiologischer Daten ist zwingend erforderlich.
- Soziale Ungleichheit und Stigmatisierung müssen bekämpft werden.
- Aufklärung und Behandlung vulnerabler Gruppen müssen verbessert werden.
- Mikro-Eliminations-Strategien sind ein kosteneffizienter und effektiver Weg zur Erkennung und Elimination von Virushepatitis in Hochrisikogruppen.
- Barrieren für einen Zugang zur Diagnostik, Prävention und Therapie für Menschen mit intravenösem Drogenkonsum müssen reduziert werden.
- Die Hinzunahme des Screenings auf eine Virushepatitis in die allgemeine Gesundheitsuntersuchung („Check-up 35“) ist eine wichtige Ressource auf dem Weg zur Elimination. Diese Gesundheitsuntersuchung muss allen offenstehen.
- Eine adäquate Nachsorge ist wichtig, um Behandlungserfolge sicherzustellen.

Die Vorträge und weitere Informationen zum Strategietreffen unter: https://www.deutsche-leberstiftung.de/veranstaltungen/veranstaltungen-der-deutschen-leberstiftung/strategietreffen-2022/
